# Effects of Perinatal Exposure to Ketamine on the Developing Brain

**DOI:** 10.3389/fnins.2019.00138

**Published:** 2019-02-22

**Authors:** Hoi Man Cheung, David Tai Wai Yew

**Affiliations:** ^1^School of Chinese Medicine, The Chinese University of Hong Kong, Sha Tin, Hong Kong; ^2^Hong Kong College of Technology, Sha Tin, Hong Kong

**Keywords:** ketamine, prenatal, neonatal, neurotoxicity, apoptosis, neurogenesis, synaptogenesis, oxidative stress

## Abstract

Initially used as an analgesic and anesthetic, ketamine has unfortunately been abused as a popular recreational party drug due to its psychotropic effects. Over the last decade, ketamine has also emerged as an effective rapid-onset anti-depressant. The increasingly widespread use and misuse of the drug in infants and pregnant women has posed a concern about the neurotoxicity of ketamine to the immature brains of developing fetuses and children. In this review, we summarize recent research findings on major possible mechanisms of perinatal ketamine-induced neurotoxicity. We also briefly summarize the neuroprotective effects of ketamine in the presence of noxious stimuli. Future actions include implementation of more drug abuse education and prevention campaigns to raise the public’s awareness of the harmful effects of ketamine abuse; further investigations to justify the clinical use of ketamine as analgesic, anesthetic and anti-depressant; and further studies to develop alternatives to ketamine or treatments that can alleviate the detrimental effects of ketamine use, especially in infants and pregnant women.

## Introduction

Ketamine is an *N*-methyl-D-aspartate (NMDA) receptor antagonist that has analgesic and anesthetic effects with rapid onset and short duration of action (Domino et al., 1965). It is well established that ketamine has low influence on respiratory and cardiac functions – unlike many other anesthetic agents, it does not induce significant respiratory depression (Domino et al., 1965; Saraswat, 2015); on the other hand, it induces transient and slight increase in heart rate, cardiac output and blood pressure instead of causing cardiac depression (Domino et al., 1965; Johnstone, 1976). The latter feature of ketamine-induced anesthesia makes the drug a favored choice especially in emergency medicine where patients with severe trauma may be at risk of hypotension.

After its approval by the Food and Drug Administration in 1970, ketamine has soon been widely used as a human anesthetic due to its safety profile and ease of use. In particular, during the Vietnam War, ketamine was the most extensively used anesthetic for injured soldiers in the battlefield. It has been listed as an anesthetic in the World Health Organization Model List of Essential Medicines since 1985. Until now, ketamine is still used in veterinary medicine as well as in pediatric practice for procedural sedation and general anesthesia (Bergman, 1999; Roelofse, 2010).

Due to its dissociative psychotropic effects, low price and increased ease of availability in the 1970s, ketamine has emerged as a popular agent of abuse on the party scenes. Furthermore, ketamine has recently been reported to possess anti-depressant effects which has rapid onset and was even effective in patients with treatment-resistant depression (Berman et al., 2000; Murrough et al., 2013). As a result, ketamine has now emerged as an off-label anti-depressant drug. In these cases, the use of ketamine in females with unexpected pregnancy may impose danger to the developing fetuses, especially to the developing brains. Table 1 summarizes the current medical and non-medical uses of ketamine. Over the last few decades, while ketamine has been used widely in clinics and abused as a popular recreational drug, at the same time, substantial evidence is showing that prenatal and early postnatal exposure to ketamine could have detrimental effects on the development of immature brains. In this article, we reviewed recent *in vitro* and *in vivo* studies and discussed some of the main findings regarding the mechanisms of the ketamine-induced neurotoxicity to the developing brains.

**Table 1:** Uses of ketamine.

Category	Description
Medical use	• Anesthesia, particularly in pediatrics, veterinary and field medicine • Pain management, at sub-anesthetic dosage • Rapid-acting anti-depressant, in patients with treatment-resistant depression
Non-medical use	• Psychotropic recreational drug

## Perinatal Exposure to Ketamine

Medical uses of ketamine on newborns and children, for instance, in pediatric anesthesia for surgical procedures and for sedation during imaging studies, involve direct application of the drug to the young, most commonly via intramuscular or intravenous injections. However, in the case of indirect administration that involves maternal transfer in clinical uses such as delivery anesthesia or recreational use, it is necessary for ketamine to move across the maternal-fetal barrier or to be released in the breast milk. The ability of ketamine to pass through the blood-placental barrier has been known for decades. In humans, ketamine levels in the cord blood of newborns reach the levels in maternal venous blood as early as 1 min 37 s after intravenous injection to healthy mothers immediately before forceps delivery (Ellingson et al., 1977). Similarly, rapid transplacental passage of ketamine has also been demonstrated in animals following intravenous administration of the drug to the dam (Craft et al., 1983; Musk et al., 2012). Mickley et al. (1998) have shown that an intraperitoneal administration of ketamine at 70 mg/kg produced a level of the drug in cerebral hemispheres of newborn rats comparable to that in fetuses at gestational day (GD) 18 after an injection of 100 mg/kg ketamine to the mother. Furthermore, a significant amount of ketamine and its metabolite norketamine could be detected in the hair samples of a neonate born to a mother suspected of abusing ketamine during pregnancy (Su et al., 2010). In lactating mice, phencyclidine, the NMDA receptor antagonist which is pharmacologically related to ketamine, crosses readily into breast milk and its concentrations were ten-fold higher than in plasma (Nicholas et al., 1982). Ketamine was also present in serum of rat littermates suckling from mothers daily treated with 3 mg/kg ketamine (Øye et al., 1993). Given that ketamine and its main active metabolite norketamine both have a preferential distribution to the brain (Cohen et al., 1973), the rapid entry of the drug to the fetal/neonatal circulation thus easily leads to an accumulation in the brain and alters the neuro-development.

## Neurotoxicity of Ketamine to the Developing Brain

The developing brain is particularly susceptible to the neurotoxicity of ketamine compared to the mature adult brain. However, the timing of key brain development and maturation processes varies between species (Semple et al., 2013). Fundamental stages of brain development which are important when considering the vulnerability to neurotoxicity include cell proliferation, neuronal differentiation, dendritic development and synaptogenesis (Semple et al., 2013). In rodents, the most common animal models in *in vivo* studies, brain growth spurt occurs postnatally during the first two to 3 weeks of life; whereas in humans, brain growth spurt starts during the last trimester of gestation and continues until 2 to 3 years of age (Dobbing and Sands, 1979; Semple et al., 2013). Disturbance in brain development during these critical periods is likely to trigger long-term brain dysfunctions and ultimately lead to neurobehavioral impairment.

### Behavioral Observations

The first animal study that reported the neurobehavioral effects of perinatal exposure to ketamine was performed in rats (Øye et al., 1993). In this study, rats that have been exposed to daily sub-anesthetic dose of ketamine (3 mg/kg) prenatally *in utero* plus postnatally through suckling showed reduced discriminative learning ability several weeks after weaning. Nevertheless, the performance of the rats exposed to ketamine prenatally only did not differ from the controls. This suggested that the blockade of NMDA receptor by ketamine at early postnatal life in rats could impair synaptic plasticity and lead to long-term deleterious effects on the retention or acquisition of conditioned behavior later in life (Øye et al., 1993). The lack of effects of prenatal treatment alone seemed to be in agreement with the critical period of synaptogenesis occurring postnatally from birth to 3 weeks of age in rodents. In another study, a single injection of high-dose ketamine (50 mg/kg) to postnatal day (PND) 10 newborn mice led to altered neurofunctional behaviors tested at 2 months of age (Fredriksson et al., 2004). These changes in neurobehaviors were manifested as deficits in habituation in the spontaneous motor activity test, dramatic impairment in acquisition learning and retention memory in the radial arm maze-learning task, and reduced shift learning in the circular swim maze-learning task (Fredriksson et al., 2004). The behavioral changes may be attributed to the enhanced neuronal degeneration in the parietal cortex detected as early as 24 h after ketamine exposure (Fredriksson et al., 2004). The two independent animal studies described above demonstrated that both ketamine treatment paradigms, whether sub-chronic at sub-anesthetic dose or a single injection at high dose, if performed during the peak developmental period, could both lead to neurobehavioral deficits later at adolescent or early adult periods.

Several other groups have also utilized behavioral procedures to evaluate the neurofunctional effects of ketamine exposure in laboratory animals. In most studies, administration of ketamine during gestational and/or neonatal periods led to neurocognitive deficits and impaired learning and memory, with the exception of the data reported by Mickley et al. revealing a beneficial effect of prenatal ketamine exposure at particular time points, which will be discussed later. Otherwise, in general, early exposure to ketamine (1) impaired the spatial learning and memory in Morris water maze (Fredriksson et al., 2004; Yan et al., 2014; Zhao et al., 2014; Zhang et al., 2016; Li X. et al., 2017; Li Y. et al., 2017), (2) induced anxiety-like behavior manifested as increased spontaneous motor activity in open field test (Fredriksson et al., 2004; Mickley et al., 2004; Aligny et al., 2014; Zhao et al., 2014), (3) suppressed fear learning and memory in fear conditioning tests (Yan et al., 2014; Zhang et al., 2016; Li X. et al., 2017; Li Y. et al., 2017), and (4) reduced motivation in force swimming test and sucrose preference test (Zhao et al., 2014). These behavioral changes were observed at adolescent or early adult ages. Paradoxically, Mickley et al. (1995, 2004) observed that conditioned taste aversion was potentiated and escape latency in water maze test was reduced in rats treated with ketamine prenatally on GD 18, indicating enhancement of learning and memory. These beneficial effects were not observed when ketamine was administered on GD 19 or PND 0 (Mickley et al., 1998, 2004), suggesting that ketamine may serve different neurochemical functions during early brain development, with GD 18 being a critical time point of the functional switch in rats. However, as these observations were reported by a sole research group, certainly further investigations will need to be performed to come to this conclusion. A summary of the effects of perinatal exposure to ketamine on rodent behavior is presented in Table 2.

**Table 2:** Summary of the effects of perinatal exposure to ketamine on rodent behavior.

Behavioral test	Related neurofunction	Period of ketamine exposure	Observation in ketamine-treated animals
Morris water maze	Spatial learning and memory	Prenatal	↑ Escape latency and ↓ probe quadrant time (Li X. et al., 2017; Li Y. et al., 2017)↑ Escape latency (Zhao et al., 2014)↓ Escape latency (Mickley et al., 2004)
		Postnatal	↑ Escape latency and ↓ probe quadrant time (Yan et al., 2014; Zhang et al., 2016)↓ Shift learning (Fredriksson et al., 2004)
Open field test	Anxiety-like behavior, locomotor activity	Prenatal	No effects (Zhang et al., 2016)↑ Locomotor activity (Mickley et al., 2004; Aligny et al., 2014; Zhao et al., 2014)↑ Vertical activity and grooming behavior; ↓ exploration in central zone (Coronel-Oliveros and Pacheco-Calderón, 2018)
		Postnatal	↑ Locomotor activity (Fredriksson et al., 2004)
Fear conditioning	Fear learning and memory	Prenatal	↓ Freezing time (Li X. et al., 2017; Li Y. et al., 2017)
		Postnatal	↓ Freezing time (Zhang et al., 2016)↓ Passive avoidance (Yan et al., 2014)
Radial arm maze	Spatial learning and memory	Postnatal	↓ Acquisition performance (Fredriksson et al., 2004)
Forced swimming test	Motivation, depression-like behavior	Prenatal	↑ Immobility time (Zhao et al., 2014; Coronel-Oliveros and Pacheco-Calderón, 2018)
Sucrose preference test	Motivation, depression-like behavior	Prenatal	↓ Sucrose preference (Zhao et al., 2014)
Conditioned taste aversion	Learning and memory	Prenatal (GD 18)	↑ Conditioned taste aversion (Mickley et al., 1995)
		Postnatal (PND 1)	↓ Conditioned taste aversion (Mickley et al., 1998)
Reward-driven discriminative task	Motivation, learning and memory	Prenatal plus postnatal	↓ Discriminative learning ability (Øye et al., 1993)
Social interaction test	Social behavior	Prenatal	Social withdrawal in adult stage; aggressive behavior since pubertal stage (Coronel-Oliveros and Pacheco-Calderón, 2018)
Spontaneous alternation on T-maze	Spatial memory and anxiety-like behavior	Prenatal	↓ Positive successes; ↑ defecation (Coronel-Oliveros and Pacheco-Calderón, 2018)

*↑: increase; ↓: decrease. GD, gestational day; PND, postnatal day.*

Putting together the data from behavioral studies described above, it is reasonable to postulate that perinatal exposure to ketamine could trigger neurochemical changes/neurodegeneration of the immature brains, which subsequently lead to dramatic disruption of functions through child development to adolescence and ultimately to adulthood. It has been hypothesized that glutamatergic dysfunction may be involved in the etiology of schizophrenia (Moghaddam et al., 1997; Frohlich and Van Horn, 2014), a neuropsychiatric disorder with peak onset periods at late adolescence and early adulthood. In this regard, Coronel-Oliveros and Pacheco-Calderón (2018) has recently proposed an animal model of schizophrenia using rats prenatally exposed to ketamine. In humans, schizophrenia is characterized by positive symptoms (e.g., hallucination, delusion), negative symptoms (e.g., loss of motivation, social isolation), and cognitive symptoms (e.g., impairments in attention and memory). Coronel-Oliveros and Pacheco-Calderón (2018) observed that the animals prenatally exposed to ketamine exhibited disinhibition and hyperactive behavior in pubertal stage, and cognitive impairments, social withdrawal, anxiety, depression, and aggressive-like behaviors in adulthood. These schizophrenia-like phenotypes in animals with prenatal ketamine challenge strongly supported the deleterious effects of ketamine on neurobehavioral functions throughout life.

To better resemble the neurobehavioral effects of ketamine in humans, extensive behavioral studies have been performed using non-human primates (Paule et al., 2011). Cognitive function tasks were performed at 3 years of age to assess aspects of learning, motivation, color discrimination and short-term memory in rhesus monkeys exposed to ketamine. Rhesus monkeys that had undergone a single intravenous ketamine anesthesia in the neonatal period displayed long-lasting significant deficits in cognitive functions, i.e., impaired learning ability, poorer color and position discrimination, and reduced motivation but no effects on short-term memory (Paule et al., 2011). In two retrospective clinical observations, the use of anesthesia at young children (below 2 or 3 years of age) was associated with disturbed neurobehavioral outcomes in late childhood or adolescence, although neither of the studies specified the anesthetic agents used (Kalkman et al., 2009; Ing et al., 2012). Taken together, these findings suggested that behavioral effects of exposure of the immature brains to ketamine during a critical period of neural development appeared to be long-term and possibly permanent.

### Enhanced Neuronal Cell Death

Over the last 20 years, *in vitro* and *in vivo* studies have both demonstrated that ketamine triggered neuronal cell death in immature brains. Ikonomidou et al. (1999) first revealed that transient blockade of NMDA glutamate receptors with ketamine during neonatal stage triggered widespread apoptotic neurodegeneration in the developing rat brains. These apoptotic effects were confined to neurons but not glial fibrillary acidic protein (GFAP)-expressing astrocytes (Ikonomidou et al., 1999). This specificity to neurons was also demonstrated *in vitro* in differentiated neural cells, where ketamine only reduced the number of polysialic acid neural cell adhesion molecule (PSA-NCAM)-positive neurons but not GFAP-positive astrocytes or O4-positive oligodendrocytes (Slikker et al., 2015). Consistent with these findings, other groups have also reported the neurotoxic effects of ketamine in developing rodents (Fredriksson et al., 2004; Scallet et al., 2004; Young et al., 2005; Liu et al., 2011; Yan et al., 2014; Gaeb et al., 2016; Pancaro et al., 2016; Zhao et al., 2016). Of note, the developmental toxicity of ketamine to immature brains depended on the dosage, duration and frequency of administration. For example, repeated doses of ketamine at 20 mg/kg injected to PND 7 rat pups resulted in a drastic increase in neuronal cell death but lower doses and/or fewer injections did not have observable effects (Scallet et al., 2004; Liu et al., 2011). In rhesus monkeys, infusion of ketamine at anesthetic dose for prolonged duration (5–24 h) caused significant neuronal damage but 3-h exposure did not (Slikker et al., 2007; Zou et al., 2009; Brambrink et al., 2012); whereas enhanced neuronal cell death was only observed after exposure to ketamine at GD 122 or PND 5 but not at PND 35 (Slikker et al., 2007). These observations further supported the notion that immature brains at earlier developmental stages are more sensitive to ketamine-induced damage. In particular, ketamine triggered greater loss of neuronal cells in the fetal (GD 120) than neonatal (PND 6) brains (Brambrink et al., 2012).

Ketamine-induced neuronal degeneration was mostly identified as apoptotic in nature. Caspase-3 is a member of the cysteine-aspartic acid protease family which is a crucial mediator of programmed cell death (apoptosis). Given its essential role in apoptotic chromatin condensation and DNA fragmentation, caspase-3 is thus important for normal brain development, as well as normal homeostasis and diseases (D’amelio et al., 2010). In the studies of ketamine-induced cell death, the immunodetection of cleaved (activated) caspase-3 is commonly used for the quantification of apoptotic cells (Scallet et al., 2004; Young et al., 2005; Slikker et al., 2007; Zou et al., 2009; Desfeux et al., 2010; Sinner et al., 2011; Brambrink et al., 2012; Liu et al., 2012; Bai et al., 2013; Aligny et al., 2014; Yan et al., 2014; Pancaro et al., 2016; Zhao et al., 2016). Terminal deoxynucleotidyl transferase dUTP nick end labeling (TUNEL) assay is also a common method for the detection of apoptotic DNA fragmentation (Ikonomidou et al., 1999; Wang et al., 2005, 2006; Sinner et al., 2011; Liu et al., 2012; Bai et al., 2013; Yan et al., 2014; Slikker et al., 2015; Gaeb et al., 2016; Zhao et al., 2016). Although there has been doubt about the accuracy of TUNEL assay to distinguish between apoptotic and necrotic cell death (Kraupp et al., 1995), most studies mentioned above reported their findings from TUNEL assay in combination with other methods that confirmed the nature of the observed ketamine-induced cell death. For instance, other than activated caspase-3 labeling, some researchers examined the degenerating cells ultrastructurally using electron microscopy and verified that the degeneration was apoptotic by the presence of nuclear condensation and fragmentation (Ikonomidou et al., 1999; Young et al., 2005; Slikker et al., 2007; Liu et al., 2011). Others evaluated markers of apoptosis such as the expression of BCL2-associated X protein (BAX), BCL2-interacting mediator of cell death (BIM), and annexin V by fluorescence-activated cell sorting (Wang et al., 2005; Desfeux et al., 2010; Soriano et al., 2010; Aligny et al., 2014; Yan et al., 2014; Slikker et al., 2015). Microarray analysis on ketamine-induced alterations in gene expression in neonatal rat brains has revealed an association between ketamine exposure and significant changes in the expression of thirty-two apoptosis-related genes suggesting increased apoptosis in developing rat brain treated with ketamine (Shi et al., 2010; Liu et al., 2011).

While most investigations focused on the measurement of apoptotic cell damage, few studies have evaluated the necrotic effects of ketamine on immature brains. Lactate dehydrogenase (LDH) is a cytoplasmic enzyme present ubiquitously in all living cells. The leakage of LDH is an indication of loss of membrane integrity and as such the measurement of LDH release has been useful for the evaluation of necrotic cell death which is accompanied by increased plasma membrane permeability (Legrand et al., 1992). Several studies have quantified the cellular LDH release upon ketamine exposure but the results were inconclusive. In neonatal rodent brain culture, treatment with ketamine did not enhance the release of LDH into the culture media (Wang et al., 2005; Desfeux et al., 2010). Similar observations have also been reported in fetal rat neural stem cells (Dong et al., 2012; Slikker et al., 2015). Furthermore, electron microscopic examination of neocortical neurons from PND 7 mice exposed to ketamine showed that the ultrastructural appearance was characteristic of apoptosis but not necrosis (Young et al., 2005). In contrast, in the frontal cortex of PND 5 monkeys exposed to ketamine, electron micrographs revealed both apoptotic (nuclear condensation and fragmentation) and necrotic (neuronal mitochondrial swelling and neuronal cell body swelling) features (Slikker et al., 2007). This is in line with the findings in frontal cortical culture from PND 3 rhesus monkeys and human embryonic stem cells, where cellular release of LDH was elevated upon exposure to high concentrations of ketamine for long durations (Wang et al., 2006; Bosnjak et al., 2012). A possible explanation is a species difference in which neuronal cells of primates are more vulnerable to the necrotic effects of ketamine than rodents. In this case, early exposure to ketamine in humans may induce more severe damages relative to laboratory rodents due to necrosis, a more detrimental form of cell death compared to apoptosis.

Taken together, there was convincing evidence to support that ketamine triggered widespread apoptotic neurodegeneration in the developing brains. However, whether ketamine induced both apoptosis and necrosis in the immature brain was still unclear and this will need to be elucidated further.

### Alterations in Neurogenesis

Neurogenesis, the process by which new functional neurons are formed, occurs most actively at developmental stages during the embryonic and early postnatal periods, although it is also sustained throughout adult life in the mammalian brain at restricted regions. During adulthood, neurogenesis is mainly identified at two locations, the subventricular zone (SVZ) of the lateral ventricles and the subgranular zone of the dentate gyrus (DG) in the hippocampus (Zhao, 2007) and is implicated in learning and memory (Zhao et al., 2008). During early developmental stages, neurogenesis is responsible for generating all various types of neurons and thus is critical to the formation of a properly functional central nervous system (CNS) throughout life. Therefore, the immature brain is particularly vulnerable to the toxicity of any agents that could alter embryonic neurogenesis and the resultant deficits in brain functions are likely long-lasting.

Ketamine at concentrations that do not trigger enhanced cell death has been shown to alter proliferation and differentiation of neural stem/progenitor cells (NSPCs). *In vitro*, ketamine at sub-apoptotic concentrations inhibited proliferation of cultured rat fetal NSPCs, assessed by reduced bromo-deoxyuridine (BrdU) incorporation into nuclei and reduced Ki67-positive immunostaining (Dong et al., 2012; Wu et al., 2014). These findings have been confirmed *in vivo* where perinatal exposure of rats to ketamine also substantially inhibited proliferation of neural stem cells (NSCs) in the ventricular zone (VZ) and SVZ, the two major regions of embryonic neurogenesis (Huang et al., 2015; Dong et al., 2016). The effect was greater in the SVZ as inhibition of proliferation was observed at lower concentrations of ketamine (Dong et al., 2016). In addition to the effects in relation to developmental neurogenesis, Zhao et al. (2014) evaluated the effects of *in utero* ketamine administration on the proliferation of neurons in DG and SVZ in rats. As mentioned earlier, these two brain areas are primary sites of adult neurogenesis and are important for synaptic plasticity (Lledo et al., 2006). The inhibition of neuronal proliferation in these regions examined at birth as well as adolescent stage (PND 30) indicated that ketamine might not only impair developmental but also adult neurogenesis (Zhao et al., 2014).

With regard to the effects of ketamine on neuronal differentiation, the results from different studies have been inconsistent. Dong et al. (2012) reported a general increase in neuronal differentiation as ketamine-treated NSPCs displayed increased labeling of Tuj-1, an early biomarker of neural cell differentiation. Similarly, maternal administration of ketamine also promoted the neuronal differentiation of NSCs in the SVZ of neonatal rats (Huang et al., 2015). However, the same study revealed an attenuation of astrocytic differentiation by ketamine, suggesting the effects of ketamine were cell type-specific (Huang et al., 2015). On the contrary, Akeju et al. (2014) showed that ketamine impaired the step-wise specification of embryonic stem cells into neuronal, neuronal progenitor, and neuroectodermal cells, observed by reduced expression of specific markers at each differentiation step.

Several studies have further delineated the possible signaling pathways that mediate ketamine-induced alterations of neurogenesis (neuronal proliferation and differentiation). These studies suggested possible involvement of the TGF-β superfamily signaling pathway, PI3K/Akt-p27 signaling pathway, and Ca^2+^-PKCα-ERK1/2 signaling pathway (Akeju et al., 2014; Dong et al., 2014; Wu et al., 2014). It is not surprising that the above signaling mechanisms might be implicated in the changes in neurogenesis triggered by ketamine, as these pathways have been known to regulate cell proliferation and differentiation (Oh and Chun, 2003; Peltier et al., 2007; Massagué and Xi, 2012). Altogether, scientific studies have clearly indicated that ketamine, at concentrations that do not induce cell loss, could still disturb normal proliferation and differentiation of neurons, thus altering neurogenesis and possibly leading to impaired brain functions.

### Disturbance in GABAergic Interneuron Development

It has been well established that in the adult brain, γ-aminobutyric acid (GABA) is the principal inhibitory neurotransmitter. The balance between inhibitory and excitatory (primarily using glutamate as neurotransmitter) synaptic transmission is pivotal in normal neuronal communication and brain functions. However, GABA-releasing neurons are formed before the glutamatergic neuron population during development (Soriano et al., 1986). It is now clear that in the immature brains where synaptic transmission is yet to emerge, GABA is, on the contrary, excitatory and has trophic effects on neuronal development (Lujan and Shigemoto, 2005; Represa and Ben-Ari, 2005; Li and Xu, 2008). Given its importance in modulating various key neuronal developmental steps, it is not surprising that disrupting the GABAergic system during early neuronal developmental stages would lead to aberrant functions of the brain. Indeed, several neurodevelopmental diseases have been associated with the dysfunction of the GABAergic system, for example, autism, schizophrenia and epilepsy (During et al., 1995; Lewis et al., 2005; Chao et al., 2010).

Prenatal blockade of NMDA receptor by the antagonist MK-801 has been shown to reduce the density of parvalbumin-positive GABAergic neurons in the medial frontal cortex of juvenile and post-pubertal rat offspring (Abekawa et al., 2007). Similarly, in immature GABAergic interneurons, incubation with ketamine at concentrations ≥10 μg/ml triggered remarkable cell death; whereas long-term exposure to ketamine at non-apoptotic concentrations (as low as 0.01 μg/ml) impaired dendritic growth and arbor development of the immature GABAergic neurons (Vutskits et al., 2006). The same research group further examined the effects of ketamine on differentiated GABAergic interneurons and showed that short-term incubation of ketamine at concentrations ≥20 μg/ml reduced cell survival; whereas at non-cell death-inducing concentrations (10 μg/ml), ketamine initiated long-term interference of the dendritic arbor architecture, including dendritic retraction and branching point elimination (Vutskits et al., 2007). In a recent study, Aligny et al. (2014) reported enhanced apoptotic death of GABAergic precursors and long-term deficits in interneuron density, dendrite numbers and spine morphology (Aligny et al., 2014). Interestingly, they further observed that the cytotoxic effect of ketamine was particularly strong in the medial ganglionic eminences and along the migratory routes of GABAergic interneurons (Aligny et al., 2014). Mature GABAergic interneurons in the cerebral cortex are derived from the ganglionic eminences located in the ventral area of the telencephalon (Kelsom and Lu, 2013). Tangential cell migration of newborn neurons from their origin to their final destination is required for the proper development of cortical GABAergic cell population (Anderson et al., 2001). The observations of Aligny suggested that ketamine may impose a long-term deficit in brain function by impairing the integration of GABAergic neurons into the cerebral cortex (Aligny et al., 2014).

### Differential Expression of NMDA Receptor

Ketamine was discovered as a chemical derivative of the NMDA receptor antagonist phencyclidine. The anesthetic, analgesic and psychotropic effects of ketamine are attributed to its antagonistic action on the NMDA receptor. As the investigations on ketamine-induced neurotoxicity continue, evidence showing that NMDA receptor was directly involved in the neurotoxic effects of ketamine has emerged. Wang et al. (2005, 2006) demonstrated that the blockade of NMDA receptor by ketamine increased cell death in both neonatal rat and rhesus monkey brain cultures, and this was associated with a compensatory up-regulation of the expression of NR1, an obligatory subunit of the NMDA receptor. More importantly, the increased cell death was blocked by antisense oligonucleotides of NR1 (Wang et al., 2005, 2006). *In vivo*, ketamine treatment to PND 7 rat pups also induced an increase in NR1 mRNA levels (Liu et al., 2011). Intriguingly, the increase of NR1 expression was highest in the frontal cortical areas where the most severe neurodegeneration was observed, and time-course analysis indicated that the neurodegenerative effect of ketamine was closely associated in time with the up-regulation of NR1 receptor subunit rather than the plasma or brain tissue ketamine levels (Liu et al., 2011). The spatial and temporal association between the ketamine-induced degeneration and NR1 up-regulation presented in these studies suggested the possibility that the up-regulation of NR1 was required for the ketamine-induced cell death.

Differential expression of other NMDA receptor subunits has also been reported. Prenatal ketamine treatment triggered an NR2A receptor subunit up-regulation and NR2B down-regulation in the hippocampus of young adult rats (Zhao et al., 2014). In contrast, in the prefrontal cortex, prenatal exposure to ketamine triggered a down-regulation of NR2A but up-regulation of NR2B (Zhao et al., 2016). The reason for the paradoxical effects of prenatal ketamine on different brain regions is currently not known, but disruption to NMDA-mediated transmission has been implicated in the pathophysiology of mood disorders (Drevets et al., 2008). Indeed, post-mortem studies of patients diagnosed with depressive disorders showed aberrant expression of NMDA receptor subunits (Karolewicz et al., 2005, 2009). In agreement with these observations in humans, alterations in the expression of NR2 subunits upon *in utero* exposure of ketamine were accompanied by anxiety- and depression-like behaviors as well as deficits in spatial memory capabilities as shown in the Morris water maze in young adult rats (Zhao et al., 2014). In global gene expression profiling studies on neonatal brain exposed to ketamine, pathway analysis identified that glutamate receptor signaling was up-regulated by ketamine, with increased expression of five glutamate receptor genes, including those encoding the NR1 and NR2 receptor subunits (Shi et al., 2010). The general compensatory up-regulation of glutamate receptors is believed to lead to over-stimulation of the glutamatergic system and the subsequent enhanced glutamate-mediated excitotoxicity, particularly in the frontal cortex (Chao et al., 2010). These findings implied that prenatal exposure of ketamine may impose neurobehavioral effects via an imbalance of NMDA receptor expression.

### Increased Oxidative Stress

Extensive evidence has suggested the contribution of oxidative stress in aging to the pathogenesis of various neurodegenerative diseases, such as Alzheimer’s disease, Parkinson’s disease, amyotrophic lateral sclerosis, and Huntington’s disease (Floyd and Hensley, 2002; Barnham et al., 2004). Compared to other internal organs, the brain is relatively prone to oxidative damage due to its high oxygen consumption, high content of unsaturated fatty acids but modest antioxidant defense, thus high rates of production of reactive oxygen species (ROS) and lipid peroxidation.

Reactive oxygen species (ROS), namely superoxide and peroxide anions as well as hydroxyl radicals, are naturally produced in normal cellular conditions during oxidative phosphorylation in the mitochondria. Other ROS are also generated in normal physiology, for example, in immune defense and redox signaling. Increased oxidative stress occurs when there is an imbalance between the production of ROS and the antioxidant defense of the biological system. The over-production of such ROS would have toxic effects by damaging cellular components such as DNA, lipids and proteins. Several studies have demonstrated that ketamine raised the intracellular levels of ROS in cultured neurons or immature brains (Bosnjak et al., 2012; Bai et al., 2013; Yan et al., 2014; Zhang et al., 2016; Li et al., 2018a,c) and these cellular changes might be mediated via the ROS/hypoxia inducible factor-1α pathway (Yan et al., 2014). As a consequence of excessive net production of ROS, perinatal exposure to ketamine also increased the contents of malondialdehyde (MDA) and 8-hydroxy-2′ -deoxyguanosine (8-OHdG), markers of oxidative insults to lipid and DNA, respectively, in the hippocampus and frontal cortex of young animals (Yan et al., 2014; Gaeb et al., 2015; Zhang et al., 2016; Li et al., 2018a,c). In contrast, the total antioxidant capacity was reduced (Li et al., 2018a,c). Not surprisingly, the ketamine-induced ROS production and the resultant apoptotic cell death could be attenuated by the addition of the ROS scavenger, Trolox (Bosnjak et al., 2012; Bai et al., 2013).

It seemed clear that oxidative stress was involved in the ketamine-induced neuronal damage in the immature brains, but where are the sources of the ROS and what causes the net increase in ROS production? Ketamine reduced the total antioxidant capacity of neurons in the hippocampus of ketamine-exposed fetuses (Li et al., 2018a,c). This may be related to the impairment of the cellular antioxidant defense, including changes in the levels of antioxidant enzymes as reported by Bosnjak et al. (2012), that ketamine triggered differential expression of glutathione synthetase, glutathione reductase, superoxide dismutase 1 and glutathione peroxidase 1, as well as two oxidative stress-related genes, oxidation resistance 1 and oxidative stress responsive 1 (Bosnjak et al., 2012). The same research group also observed that ketamine stimulated over-production of ROS with depolarization of mitochondrial membrane potential and the release of cytochrome c from mitochondria, suggesting the neurotoxicity was mediated via a mitochondrial pathway (Bosnjak et al., 2012; Bai et al., 2013).

More recent studies, however, suggested that the ketamine-induced oxidative stress in the developing brain was not derived from mitochondria. Zhang et al. (2016) reported no differences in the electron transport chain enzymatic activities of complexes I and III and ATP levels in the hippocampal mitochondrial fractions after repeated neonatal ketamine exposures in rats. Instead, ketamine treatment stimulated an up-regulation of the superoxide-generating enzyme, NADPH oxidase 2 (NOX2), which in turn caused loss of phenotype of parvalbumin-positive interneurons in the hippocampus and prefrontal cortex and ultimately led to long-term cognitive impairments (Zhang et al., 2016). The absence of ketamine-induced increase in 8-OHdG levels in the prefrontal cortex in NOX2-knockout mice supported the role of NOX2 as the source of ROS upon exposure to ketamine (Sorce et al., 2010). It is noteworthy that this NOX2-mediated oxidative stress led to an immediate release of the neurotransmitters, glutamate and dopamine, which may explain the schizophrenia-like behavioral alterations in response to ketamine (Sorce et al., 2010). Treatments with either the NADPH oxidase inhibitor, apocynin, or the antioxidant bioflavonoid, morin, attenuated the ketamine-induced anomalies in neurochemical profiles and behaviors (Zhang et al., 2016; Ben-Azu et al., 2018). Altogether, these findings indicated the involvement of NOX2-mediated oxidative stress in ketamine-induced neurotoxicity.

### Alterations in Developmental Synaptogenesis

Synapses are a structure that allow communication between neurons. The proper formation of functional synapses enables the establishment of neuronal circuitries, which are critical to normal cognitive and mental development. Mice subjected to a 5-h anesthesia by ketamine at PND 15 during the peak of synaptogenesis displayed increased dendritic spine density in the somatosensory cortex and hippocampus (De Roo et al., 2009). As dendritic spines serve as the postsynaptic component of synapses, the modulation of dendritic spine density by ketamine implied that the drug increased the complexity of cortical synaptic networks and thus promoted synaptogenesis. Interestingly, these findings were not only demonstrated in mice treated with ketamine, an anesthetic that blocks NMDA receptor-mediated excitation, but also with midazolam, an anesthetic that enhances GABA_A_ receptor-mediated inhibition (De Roo et al., 2009). These observations suggested that the dendritic spine growth and formation of functional synapses were not mediated by specific receptors. Instead, they were determined by the balance between excitatory and inhibitory neuronal signaling. However, results from more recent studies did not all coincide with the findings reported by De Roo. Consistent with De Roo’s findings, maternal administration of ketamine (at GD 14) led to more branched pyramidal neurons and longer basilar dendrites with increased spine density in laminae II and III of the prefrontal cortex in adolescent rats (Zhao et al., 2016). Nevertheless, in the CA1 and CA3 regions of hippocampus, the pyramidal cells exposed to ketamine exhibited the opposite profiles - the pyramidal dendrites were less branched, total branch length was shorter than in controls and spine density also decreased (Zhao et al., 2014; Li X. et al., 2017; Li Y. et al., 2017). The discrepancies in the effects of ketamine on dendritic growth presented in these studies could have been due to regional differences as well as the timing of ketamine exposure, i.e., whether ketamine was administered at mid-gestation during early brain development or postnatally at peak of synaptogenesis.

A functional synapse requires a highly regulated interplay between various molecules at the synaptic junction. In the developing brains, ketamine exposure down-regulated the expression of three synapse-associated proteins, synapsin (SYN), synaptophysin (SYP), and postsynaptic density-95 (PSD-95) (Sinner et al., 2011; Zhao et al., 2014, 2016; Li Y. et al., 2017), although one study revealed an up-regulation of PSD-95 in the prefrontal cortex of PND 30 rats (Zhao et al., 2016). SYN and SYP are two presynaptic proteins that regulate synaptic vesicle trafficking and recycling (Bloom et al., 2003; Kwon and Chapman, 2011); whereas PSD-95, as its name indicates, is a scaffolding molecule located in the postsynaptic density of neurons and is involved in the clustering of various receptors, ion channels and signaling proteins within the dendritic spine (Kornau et al., 1995). Changes in the levels of these synaptic proteins would disturb synaptogenesis and thus synaptic transmission. Additionally, it has also been reported that maternal administration of ketamine reduced the expression level of the brain-derived neurotrophic factor (BDNF) in the hippocampus at postnatal day 30 (Zhao et al., 2014; Li X. et al., 2017). This neurotrophin is involved in multiple aspects of synaptogenesis, from neuronal survival to synapse stabilization (Causing et al., 1997). Signaling pathways that require the activation of ERK, PKA, and CREB have been postulated to be involved in this ketamine-induced reduction of BDNF, which in turn altered synaptogenesis and ultimately led to cognitive impairment (Sinner et al., 2011; Li X. et al., 2017; Li Y. et al., 2017). Table 3 summarizes the major possible mechanisms of ketamine-induced neurotoxicity to the developing brain discussed in this review.

**Table 3:** Summary of major possible mechanisms of ketamine-induced neurotoxicity to the developing brain.

Mechanism	Description
Enhanced neuronal cell death	• ↑ Apoptosis (Ikonomidou et al., 1999; Scallet et al., 2004; Wang et al., 2005, 2006; Young et al., 2005; Slikker et al., 2007, 2015; Zou et al., 2009; Desfeux et al., 2010; Shi et al., 2010; Soriano et al., 2010; Liu et al., 2011, 2012; Sinner et al., 2011; Brambrink et al., 2012; Bai et al., 2013; Aligny et al., 2014; Yan et al., 2014; Gaeb et al., 2016; Pancaro et al., 2016; Zhao et al., 2016): specific to neurons, but not to astrocytes nor oligodendrocytes (Ikonomidou et al., 1999; Slikker et al., 2015) • ↑/- Necrosis: observed only in neural cells of primate origin (Wang et al., 2006; Slikker et al., 2007; Bosnjak et al., 2012), but not of rodents (Wang et al., 2005; Young et al., 2005; Desfeux et al., 2010; Dong et al., 2012; Slikker et al., 2015)
Alterations in neurogenesis	• ↓ Proliferation of neural stem/progenitor cells at sub-apoptotic concentration (Dong et al., 2012, 2016; Wu et al., 2014; Zhao et al., 2014; Huang et al., 2015) • ↑ Neuronal differentiation (Dong et al., 2012; Huang et al., 2015); ↓ neuronal differentiation (Akeju et al., 2014) • Possible signaling pathways involved: TGF-β, PI3K/Akt-p27, and Ca^2+^-PKCα-ERK1/2 (Akeju et al., 2014; Dong et al., 2014; Wu et al., 2014)
Disturbance in GABAergic interneuron development	• ↑ Cell death of immature GABAergic interneurons (Vutskits et al., 2006, 2007; Aligny et al., 2014) • ↓ Dendritic growth and arbor development (Vutskits et al., 2006, 2007; Aligny et al., 2014)
Differential expression of NMDA receptor	• ↑ Expression of the NR1 subunit of NMDA receptor (Wang et al., 2005, 2006; Shi et al., 2010; Liu et al., 2011) • ↑ NR2A expression and ↓ NR2B expression in hippocampus (Zhao et al., 2014); ↓ NR2A expression and ↑ NR2B expression in prefrontal cortex (Zhao et al., 2016)
Increased oxidative stress	• ↑ ROS (Bosnjak et al., 2012; Bai et al., 2013; Yan et al., 2014; Zhang et al., 2016; Li et al., 2018a,c) via the ROS/hypoxia inducible factor-1α pathway (Yan et al., 2014) • ↑ MDA and 8-OHdG, indicating oxidative damage to lipid and DNA, respectively (Yan et al., 2014; Gaeb et al., 2015; Zhang et al., 2016; Li et al., 2018a,c) • ↓ Total antioxidant capacity (Li et al., 2018a,c) • Source of ROS over-production: • Mitochondrial dysfunction (Bosnjak et al., 2012; Bai et al., 2013); and/or • Up-regulation of the superoxide-generating enzyme, NOX2 (Sorce et al., 2010; Zhang et al., 2016; Ben-Azu et al., 2018)
Alterations in developmental synaptogenesis	• Dendritic spine density: (postnatal exposure) ↑ in somatosensory cortex and hippocampus (De Roo et al., 2009); (prenatal exposure) ↑ in prefrontal cortex (Zhao et al., 2016) but ↓ in CA1 and CA3 of hippocampus (Zhao et al., 2014; Li X. et al., 2017; Li Y. et al., 2017) • Differential expression of the synapse-associated proteins, SYN, SYP and PSD-95 (Sinner et al., 2011; Zhao et al., 2014, 2016; Li Y. et al., 2017) • ↓ Expression of the neurotrophin, BDNF (Zhao et al., 2014; Li X. et al., 2017)

*↑: increase; ↓: decrease; -: no change.*

## Neuroprotective Effects of Ketamine

Although animal studies have demonstrated that ketamine could induce neuronal degeneration and disturb neuronal development in the immature brains, the evidence that showed the neuroprotective effects of ketamine should not be overlooked, especially in the presence of other stresses. In ischemia and traumatic brain injuries, extracellular glutamate levels were elevated (Benveniste et al., 1984; Palmer et al., 1993). These changes contribute to the excessive NMDA glutamate receptor activation that leads to excitotoxic neuronal cell death and eventually brain damage. Reduction in neuronal loss in these conditions after treatment with NMDA receptor antagonists has been reported in animals (Church et al., 1988; Spandou et al., 1999; Puka-Sundvall et al., 2000), indicating the protective effects of transient NMDA blockade against excitotoxic neuronal damage. It has also been established that ketamine has anti-inflammatory effects, which are attributed to the ability of ketamine to suppress the activity of macrophages (Chang et al., 2005, 2016; Li et al., 2018b) and to inhibit the production of pro-inflammatory cytokines, such as tumor necrosis factor-α, interleukin-6 and interleukin-1β (Beilin et al., 2004; Shaked et al., 2004; Chang et al., 2005; Gundogdu et al., 2016; Li et al., 2018b). In addition, where ketamine is used for pediatric anesthesia/analgesia, the pre-term or diseased infants are usually subjected to procedural pain and surgery-related psychological stress. These noxious stimuli and stresses occurring during a stage of active brain growth have been shown to exert detrimental neurobiological effects to the immature brain which were associated with impaired brain development and long-term behavioral consequences (Graham et al., 1999; Brummelte et al., 2012). With regard to the pain-induced neuronal damage, the use of ketamine anesthesia/analgesia appeared to attenuate the neuronal excitation and cell death in cortical and subcortical areas triggered by inflammatory pain in neonatal rats, with concomitant improved performance in cognitive test (Anand et al., 2007). Liu et al. (2012) confirmed that neonatal use of ketamine triggered neuroapoptosis, but emphasized that in the presence of painful stimuli, the ketamine-induced cell death was, however, attenuated. Altogether, these findings justified the use of ketamine for analgesia and anesthesia and supported that ketamine may be neuroprotective in the presence of pain and stress, as opposed to its neurodegenerative effects.

## Conclusion

This review has brought together the molecular and behavioral studies that demonstrated the neurotoxic effects of perinatal exposure to ketamine on the immature brains and the resultant behavioral outcomes, and also discussed several possible mechanisms by which ketamine may exert its detrimental effects. We also briefly described the neuroprotective effects of ketamine in the presence of noxious stimuli such as inflammation, pain and stress. Based on these existing findings, future investigations will be required to determine whether ketamine is safe to be used in pediatric anesthesia and as an anti-depressant drug for pregnant women. In cases where ketamine use is necessary, will there be treatments which can be given prior to or in conjunction with ketamine to mitigate its neurotoxicity? Or perhaps alternatives should be developed to replace the use of ketamine? The public sectors and local communities should also consider increasing the delivery of education and prevention programs to raise the public’s awareness of risks of ketamine abuse, especially in pregnant women.

## Author Contributions

HC wrote the manuscript. DY provided the guidance on the writing and approved the manuscript.

## Conflict of Interest Statement

The authors declare that the research was conducted in the absence of any commercial or financial relationships that could be construed as a potential conflict of interest.
